# It all depends on which side of the fence you are standing: agent and recipient perspectives are differently linked with job crafting

**DOI:** 10.1186/s40359-023-01135-0

**Published:** 2023-04-04

**Authors:** Marta Roczniewska, Magdalena Marszałek

**Affiliations:** 1 LIME Department, Karolinska Istitutet, Stokholm, Sweden; 2 SWPS University of Social Sciences and Humanities, Sopot Campus, Sopot, Poland

**Keywords:** Job crafting, Proactivity, Dual Perspective Model, Agent, Recipient

## Abstract

**Background:**

In social contexts, people may view themselves as agents, who are in control of the environment, or recipients, who succumb to what others have decided. Here, we investigated how these perspectives determine job crafting (JC)—self-initiated employee behaviors targeted at altering job characteristics to fit them with one’s needs.

**Methods:**

Study 1 tested the relationships between chronic agent–recipient tendencies and JC in a cross-lagged panel design. Study 2 was a randomized experiment where agent–recipient perspectives were manipulated to predict JC intentions in the week to follow.

**Results:**

Supporting our predictions, while agents sought structural job resources and increased challenging demands, recipients resorted to reducing hindering demands (Study 1). Study 2 revealed that activating an agent perspective led to stronger intentions to increase structural job resources and challenging demands.

**Conclusions:**

We conclude that agent and recipient perspectives are linked with differential patterns of JC behaviors. Strengthening agency is a vital step in forming job redesign goals during JC interventions.

**Supplementary Information:**

The online version contains supplementary material available at 10.1186/s40359-023-01135-0.

## Introduction

Most jobs are initially designed by organizations and the employees are the recipients of this top-down job design process. Employees may, however, *craft* their jobs, i.e., modify the job characteristics to make the jobs better fit their needs and preferences [1, 2]. This way, the job is re-designed bottom-up and employees take over the agency in the process. Given the growing body of research demonstrating positive consequences of job crafting (JC) [3], organizational stakeholders are interested to understand which conditions make JC more likely and which employees have the propensity to engage in job redesign. What we know is that higher levels of autonomy at work [4] or certain leadership styles [5] facilitate JC. However, context variables may take time or be difficult to alter due to organizational complexity. Past research has pointed to the role of proactive personality [6, 7] as an antecedent of crafting attempts. Yet, there are two problems with this predictor. First, personality is a relatively stable characteristic and thus, proactive personality would need to be included in the employee selection process. Second, meta-analysis of research on JC predictors [7] showed that proactive personality is linked with both beneficial and maladaptive crafting attempts. From research and practice perspective, we lack a predictor that is conceptualized both as a trait, but also a state that can be activated to facilitate only productive forms of crafting. It would be especially beneficial to identify a mindset that can be activated with simple and quick techniques that can be incorporated in the workplace or during interventions.

This purpose could be served by applying the Dual Perspective Model (DPM) proposed by Abele and Wojciszke [8]. It argues that a fundamental feature of any social context is the presence of two perspectives: the agent and the recipient. The perspective of the agent is taken by the one who performs an action and exerts control over the situation, whereas the perspective of the recipient is taken by the one who experiences the consequences of an agent’s actions. We propose that a tendency to assume that one is the agent in social situations predisposes individuals to be proactive in job re-design in general. When people who view themselves as agents experience a misfit with their jobs, they assume that they have the power to influence their conditions, which may contribute to introducing changes to one’s job. Those, who believe that they are only recipients of the decisions and events in the work context, will be reluctant to take a proactive stance towards their job design or may engage in a different pattern of re-design attempts. Here, we propose that agents and recipients choose to craft their jobs using different strategies that correspond to their mindsets.

The purpose of the present article is to integrate the DPM [8] with the Job Demands-Resources (JD-R) model [9] to better explain *who* engages in job crafting and *in what form*. We aim to contribute to the literature and practice in three important ways. First, by acknowledging that people may differ in their chronic tendency to assume the agent or recipient perspective, we explain why only some individuals perceive JC as an option when they are faced with misfit to their job characteristics. Second, our research provides patterns of typical JC strategies for agents and recipients, and thus, refines our understanding of *who* crafts *in what way.* This is relevant, as choosing a strategy that fits with individual preferences, rather than engaging in each type of job crafting, may be more effective in achieving the desired aim. Third, to the best of our knowledge, the DPM [8] has not been previously used to predict workplace outcomes; yet, this model explains perceptions, emotions and behaviors in social interdependence context, and as such it applies to the workplace. Because past research identified that the perspectives of agent and recipient can be temporarily activated [10], there is an opportunity to apply this framework and to design an evidence-based activity during JC training interventions as a technique to support forming JC intentions.

### Job crafting and its types

Traditionally, job design has been perceived as a top-down process wherein the job is designed and redesigned (if needed) by the organization [2]. However, the fast-paced changes that occur within and outside organizations, as well as individual differences of the employees who perform the jobs, make it difficult for organizations to create optimal job designs: one size does not fit all. Thus, JC may be a method to accommodate employees’ unique needs. JC is a form of proactive behavior that involves employees actively changing distinct aspects of their jobs. Wrzesniewski and Dutton [1] argued that employees enhance the meaning of their work through task (changing the number, scope, or type of task), relational (modifying the nature of interactions with others), and cognitive crafting (changing how one perceives their role at work). Tims and Bakker [2] framed JC in the context of the JD-R model [9] to describe changes that individuals make in their work characteristics: *job demands* (aspects of the job that result in physiological or psychological costs) or *job resources* (aspects that help reduce the strain associated with job demands, as well as are functional in achieving work goals and stimulating employee growth [11]). Based on the JD-R model, Tims et al. [6] distinguished four job-crafting strategies. First, individuals increase structural job resources (design aspects of a job, like learning opportunities or autonomy), e.g., by expanding their job discretion. Second, employees engage in increasing social job resources, e.g., by reaching out for support from colleagues. Third, employees increase the level of challenging demands by, e.g., adding more stimulating tasks. Finally, hindering job demands may be reduced to decrease the strain; for example, individuals may look for ways to minimize taxing tasks.

Integrating these role- and resource-based crafting approaches, Lichtenthaler and Fischbach [12] distinguished between crafting aimed at reaching gains (promotion crafting), versus that concerning avoiding negative end-states (prevention crafting). Similarly, Laurence [13] differentiated between expansion- and contraction-oriented JC. These streams of research on JC have been synthesized by Zhang and Parker [4] who proposed a three-level hierarchical structure of JC. The first level relates to its orientation: approach (enriching and expanding) versus avoidance (reducing and limiting) crafting. The second describes the JC form, i.e., behavioral (changes in actions) versus cognitive (changes in perceptions). The third level concerns the content of JC: altering the levels of job resources or job demands. For instance, increasing social job resources represents an approach-, behavioral-, and resources-focused crafting attempt.

Approach and avoidance crafting differ both conceptually as well as empirically. The meta-analysis by Rudolph et al. [7] showed that the dimensions of approach crafting (i.e., seeking resources and seeking challenges) and avoidance crafting (i.e., decreasing demands) have very weak relationships with each other. Also, empirical studies and meta-analytic results have shown distinct antecedents and outcomes of approach and avoidance crafting [4]. For example, approach and avoidance crafting have opposite effects for work engagement, job satisfaction, burnout, strain, and job performance [7].

### Predictors of job crafting

Certain contextual factors reinforce JC. Quantitative meta-analysis underlined the role of autonomy and workload as positive predictors of approach-oriented JC; these factors were, however, unrelated to avoidance-oriented JC [7]. A meta-synthesis of qualitative research refined the role of workload as representing a reactive motive for JC, i.e., related to the need to cope with adversity, rather than a proactive one, describing situations where individual’s initiate JC to reach desirable goals [14]. Other relevant factors include supportive work environment that stimulates and encourages JC efforts, such as high social support, a proactive organizational culture [15], or a shared organizational identity. These contexts enable both proactive and reactive motives to approach crafting. In contrast, constraining contexts (e.g., excessive supervision) prompt avoidance crafting driven by reactive and proactive motives.

Several individual differences factors predict employee crafting attempts. High proactive personality and high conscientiousness [7] are linked with general JC (i.e., aggregated scores across the dimensions). Research has demonstrated that individual differences affect the orientation of JC. Employees high in approach temperament and promotion focus tend to engage more in approach crafting, while avoidance temperament and prevention focus predict avoidance crafting [7]. There is additional variation in JC content depending on individual differences. For instance, increasing social job resources is characteristic of individuals high in extraversion or narcissism, whereas higher levels of psychoticism inhibit these attempts [16].

Knowledge of factors that promote JC in the organization is relevant given the documented benefits of JC for individual employees, work teams, and whole organizations [4]. Yet, the context factors are not always possible to be modified (i.e., workload in health care) or they take time to change (e.g., organizational culture). Individual differences that relate to temperament or personality traits are relatively stable [17] and thus, while they may serve as criteria in employee selection process, they cannot be changed in interventions that aim at boosting JC. As an alternative, JC facilitators may turn to mindsets, perspectives, or orientations, which may have stable components, but are also possible to be activated and shaped. One such possibility is presented by the Dual Perspective Model (DPM) [8].

### The agents and recipients of the social world

DPM [8] proposes that there are two major perspectives that people undertake in the social world: agent and recipient. *Agent* is the one who takes an action, and *recipient*—the one at whom the action is directed and who experiences its outcomes. This division delineates their differences in the mindset: of either being an actor, who shapes the environment, or the one who is at the receiving end of whatever takes place in the social world. An agentic mindset is linked with higher self-esteem, self-efficacy, and more positive emotions than that of a recipient [8]. Recipients focus on understanding the social world around them. This focus results in increased focus on one’s emotions and higher accessibility of communal content, i.e., traits that are relevant for social relations, like helpfulness or honesty, and higher interest in other people [18]. The foundation of the DPM is a body of research on agency and communion as two basic dimensions in social cognition (for a review, see [8]). When performing an action, one must monitor their effectiveness. Thus, taking the agent perspective involves the agency dimension. In the recipient perspective, people concentrate on the actions that affect them; therefore, they need to be vigilant towards the social value of others’ actions and their intentions. Thus, recipient perspective orients an individual to communion.

People differ in the chronic tendency to view themselves as agents or recipients [18]. The propensity to take the agent perspective is defined as a ‘habitual preference to take action, influence others and have control over the situation’ (p. 72) [10]. Chronic recipient perspective is a habitual preference to succumb to what others have decided and to withdraw from action [18]. However, certain situations naturally place individuals into one of these roles, such as when one is the physician and the other–the patient. Thus, the agent and recipient perspectives may be temporarily activated given the context and roles dedicated to one in an interaction [10]. Thus, we could speak both about chronic (trait) and temporal (state) agent-recipient perspectives.

JC requires personal initiative and as such is predicted by proactive personality, which may appear similar to DPM. The propensity to take the agent perspective is a habitual preference to view oneself as being in control and the one shaping the outcomes in a dyadic interaction. Thus, in agent-recipient perspective one usually considers themselves in relation to somebody else. Proactivity, on the other hand, refers to ‘self-initiated and future-oriented action that aims to change and improve the situation’ (p. 636) [19]. Thus, proactive behavior involves taking control and causing change, and acting in advance of a future situation. Additionally, proactivity usually refers to a sense of control over oneself and introducing change to one's life, while propensity to take the agent perspective relates not only to beliefs about control over oneself, but most importantly to behavioral tendencies and to adopting certain roles in social situations. Overall, propensities to take the agent vs recipient perspective are, thus, a wider construct which are not only related to the anticipatory self-initiated actions and personal initiative but also to particular behavioral preferences, sense of control, and autonomy in social context. Thus, we predict that these perspectives explain engaging in JC over and above proactivity. We expect that differences in agent-recipient perspectives affect not only the orientation of crafting attempts (approach versus avoidance), but also its content (i.e., types of resources and demands). Thus, the role of agency and communion concerns may provide us with a nuanced understanding of the content of JC attempts.

### Predicting job crafting patterns from dual perspectives model

The differences in the agent-recipient perspectives are related to a set of cognitive and emotional changes that occur in people’s mindsets. The agent experiences an increase in personal control and efficacy, accompanied by high positive affect and self-esteem [8]. These outcomes may also act as positive personal resources enabling an individual to craft his or her job [4]. Numerous studies inspired by the DPM have shown that agentic content is associated with the propensity to act and ‘take charge’. Abele and Wojciszke [8] propose that the agent perspective relates to a general expansion. This corresponds with approach crafting, i.e., seeking resources and challenging job demands. However, because past research showed links between agency and inhibition of communal content [20], we expect that a chronic agent perspective is linked with seeking structural resources as well as seeking challenging job demands, but not with seeking social job resources.

#### Hypotheses 1

An agent perspective is positively linked with seeking structural job resources.

#### Hypothesis 2

An agent perspective is positively linked with seeking challenging job demands.

A recipient perspective signifies being subjected to the actions of others and depending on them. Therefore, a recipient is concerned with communal content and experiences increased feelings of vulnerability [8]. Taking this perspective results in an increased interest in the social aspects of the world and one’s emotions. Past research has shown that individuals with strong interest in social contact (i.e., high in extroversion or narcissism), craft their jobs by seeking social resources [16]. Since recipients look up to others, they may be more interested in seeking their support. For JC, this would translate into pursuing more social job resources. On the other hand, the fact that the recipient perspective is related to withdrawal from actions, it could signify lack of any crafting attempts. In this study we wish to test a hypothesis that:

#### Hypotheses 3

A recipient perspective is positively linked with seeking social job resources.

Abele and Wojciszke [8] argue that the recipient mindset may result in a general contraction—a lowered tendency to act, to contribute, and to take control. Faced with problems, recipients resort to avoidance as they perceive problems as threats rather than challenges. Previous research demonstrated that chronic recipient perspective is correlated with neuroticism [18], and the latter predicts reducing demands [7]. Therefore, a propensity to a recipient role may result in reducing demands that are perceived as hindrances. On the other hand, obstacles are rather viewed as a challenge than a threat in the case of agents [8]. Overall, we predict that these two perspectives are linked with a different pattern of prevention-oriented crafting:

#### Hypothesis 4

An agent perspective is negatively linked with decreasing hindering job demands.

#### Hypothesis 5

A recipient perspective is positively linked with decreasing hindering job demands.

**Fig. 1:**
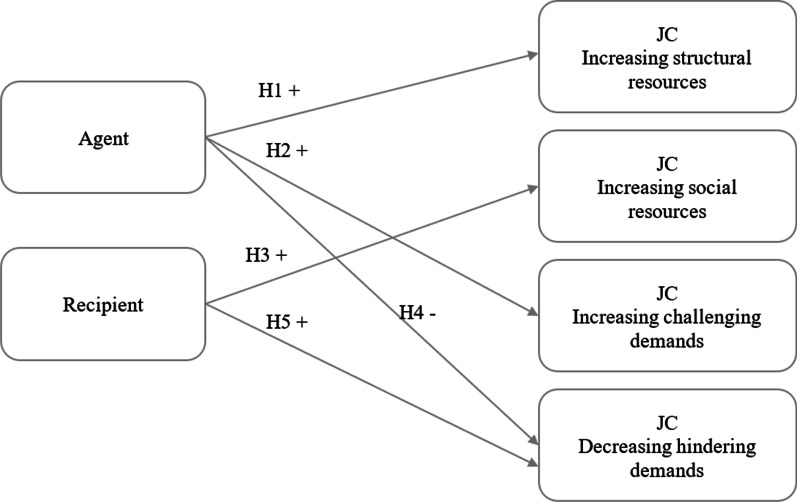
Expected relationships between agent-recipient perspectives and job crafting (JC) types

Figure 1 demonstrates the expected relationships between agent-recipient perspectives and four types of JC.

## Study 1

Study 1, a two-wave cross-lagged online study, aimed to investigate the relationships between the disposition to take agent and recipient perspectives, and four types of JC. The data that support the findings of this study are openly available in Open Science Framework at https://osf.io/a6fp9/?view_only=7390b1210f224a209e762e5d6b670cc3.

### Method

#### Power analysis

We used G*power [21] to estimate the appropriate sample size with 80% power and alpha of 0.05. We expected effect sizes in a range between small-to-medium, and thus we set f^2^ to 0.08. The statistical test was a linear multiple regression with three predictors (step 1—proactivity; step 2—agent and recipient perspectives), and the power analysis was conducted for fixed model with expected R^2^ increase for the two tested predictors in step 2. A-priori sample size calculation estimated sample size at 124. Because we designed a two-step procedure and expected a drop in participation between the measurement points, we aimed to recruit twice as many individuals in the first phase (c. 250 persons).

#### Participants and procedure

The participants were recruited through a university participant pool. The university has 5 campuses across Poland, and thus individuals were spread across the country. The questionnaire was only sent to persons who were tagged in the database as employed. There were two measurement points (T1 and T2) separated by 5–7 weeks, and the respondent’s answers were matched using a unique identifier. At T1, 324 individuals participated in the survey (267 women, 55 men, and 2 ‘other’; *M*_age_ = 29 years; *SD* = 9). At T2, the sample was *N* = 146 (125 women, 21 men; *M*_age_ = 32, *SD* = 9). The purpose of the research and the respondent’s rights were presented on the first page of the survey at both timepoints. At T1, participants completed three questionnaires: to measure proactivity, the agent and recipient perspectives, and JC. At T2, participants only reported their JC behaviors.

#### Measures

*Agent and recipient* perspectives were measured with Perspectives Questionnaire–PQ [18], which comprises 10 items measuring agent (e.g., “I like to make decisions”, “I like to have an influence on what is happening” [*α* = 0.82]), and 10 items measuring recipient (e.g., “I like to delve into my feelings”, “I really care about what other people are doing” [*α* = 0.87]) perspective. The respondents indicated how much they agreed with each item (1 = definitely not, 7 = definitely yes).

*Proactivity* was measured using a subscale of a General Proactivity Scale developed and validated by Banka [22]. This subscale consists of 9 items (e.g., “I keep looking for new ways to increase my chances of development”, “Wherever I am, I try to be a constructive force for favorable changes” [*α* = 0.87]). The respondents indicated their agreement with each statement (1 = I completely disagree, 7 = I completely agree).

*Job crafting* was measured with the Polish version [23] of the Job Crafting Scale [6]. It consists of four subscales: increasing structural job resources is (e.g., “I try to develop myself professionally” [T1: *α* = 0.79, T2: *α* = 0.83]), increasing social job resources (e.g., “I ask others for feedback on my job performance” [T1: *α* = 0.76, T2: *α* = 0.77]), increasing challenging job demands (e.g., “When an interesting project comes along, I offer myself proactively as project co-worker” [T1: *α* = 0.86, T2: *α* = 0.89]), and decreasing hindering job demands (e.g., “I try to ensure that my work is emotionally less intense” [T1: *α* = 0.74, T2: *α* = 0.76]). The participants assessed how often they engaged in each of the behaviors during the last month (1 = never/almost never, 5 = always/almost always).

### Result and discussion

#### Analysis strategy

To test the construct validity of the measures, we conducted a series of confirmatory factor analyses (CFA) (see Additional file 1). To test Hypotheses 1–5 we performed hierarchical regression analyses in two steps. To examine whether the agent-recipient perspectives explained additional variance in JC over and above proactivity, we entered proactivity in the first step. In the second step, we entered agent and recipient perspectives. We report the results of the frequency of four JC types as outcomes measured at T1 and at T2 to compare cross-sectional and cross-lagged results. Descriptive statistics can be found in Additional file 2.

#### Hypotheses testing

Table 1 demonstrates the results of the hierarchical regression analyses for the cross-sectional (T1) and cross-lagged (T2) relationships between agent and recipient perspectives and the four types of JC behaviors, controlling for proactivity. As Table 1 shows, proactivity was a positive predictor of all JC dimensions for both cross-sectional and cross-lagged links. Adding agent and recipient perspectives allowed to explain additional variance in increasing structural job resources in a cross-sectional (5%) and a cross-lagged (3%) model. In line with Hypothesis 1, agent perspective was positively linked with increasing structural job resources for both cross-sectional (*β* = 0.28) and cross-lagged (*β* = 0.19) links. Recipient perspective was unrelated to this dimension.

**Table 1:** The cross-sectional (T1) and cross-lagged (T2) relationships between agent and recipient perspectives and job crafting dimensions

	Increasing structural job resources								Increasing social job resources								Increasing challenging job demands								Decreasing hindering job demands							
	T1				T2				T1				T2				T1				T2				T1				T2			
	Model 1		Model 2		Model 1		Model 2		Model 1		Model 2		Model 1		Model 2		Model 1		Model 2		Model 1		Model 2		Model 1		Model 2		Model 1		Model 2	
	β	*p*	β	*p*	β	*p*	β	*p*	β	*p*	β	*p*	β	*p*	β	*p*	β	*p*	β	*p*	β	*p*	β	*p*	β	*p*	β	*p*	β	*p*	β	*p*
Proactivity	.47	< .001	.31	<.001	.44	<.001	.34	<.001	.19	<.001	.23	.001	.35	<.001	.39	<.001	.38	<.001	.23	<.001	.38	<.001	.28	.003	.09	.101	.16	.016	.07	.397	.19	.053
Agent			.28	<.001			.19	.036			− .07	.293			− .08	.386			.25	<.001			.19	.048			− .15	.022			− .25	.012
Recipient			− .04	.466			− .09	.224			.05	.339			.06	.422			− .04	.486			− .06	.437			.28	<.001			.30	<.001
*R*^2^ (corrected)	.22		.26		.19		.21		.03		.03		.11		.11		.14		.17		.14		.16		.01		.09		− .002		.10	
Δ*R*^2^			.05				.03				.01				.01				.04				.03				.09				.12	
*F*			10.83				2.75				0.99				0.64				7.52				2.15				16.48				9.39	
*p*			<.001				.067				.372				.532				.001				.120				<.001				<.001	

*N*_T1_ = 324, *N*_T2_ = 146

Proactivity, agent perspective, and recipient perspective were measured at T1

Model 1 represent cross-sectional relationships between the predictors and the outcomes. Model 2 represents cross-lagged relationships between predictors measured at T1 and outcomes measured at T2

Supporting Hypothesis 2, agent perspective was positively linked with increasing challenging job demands in a cross-sectional model (*β* = 0.25); in a cross-lagged model the increase in explained variance was not statistically significant (*p* = 0.120) and the agent perspective was weakly related to increasing challenging demands (*β* = 0.19). There is no support for Hypothesis 2 in cross-lagged data.

The agent-recipient perspectives did not explain additional variance in increasing social job resources in a cross-sectional or in a cross-lagged model. Contrary to Hypothesis 3, recipient perspective was not significantly linked with this type of JC.

Finally, supporting our expectations, adding the agent-recipient perspectives explained additional variance over and above proactivity in decreasing hindering job demands in both a cross sectional (9%) and a cross-lagged (12%) model. In line with Hypothesis 4, agent perspective was negatively related to decreasing hindering job in a cross-sectional (*β* = − 0.15) and in a cross-lagged (*β* = − 0.25) model. Supporting Hypothesis 5, a recipient perspective was positively linked with decreasing hindering job demands both in a cross-sectional (*β* = 0.28) and in a cross-lagged (*β* = 0.30) model.

To summarize, we found support for most of our hypotheses concerning agents: individuals with stronger propensity to take this perspective declared engaging in increasing structural job resources and in increasing challenges more frequently. The cross-lagged links were weaker than the cross-sectional links. We did not find a link between the propensity to take recipient perspective and seeking social job resources. Finally, we observed an expected pattern for decreasing hindering job demands: agents were less likely, and recipients—more likely to engage in these behaviors.

## Study 2

Overall, Study 1 supported some of our hypotheses concerning the differential relationships between the dispositional tendencies to adopt the agent-recipient perspectives and patterns of JC behaviors. However, the design of this study did not allow us to make causal inferences about the *influence* of agent and recipient perspectives on JC. Yet, this is important because establishing a possibility to increase JC behaviors and intentions to engage in them by taking the agent perspective can form the basis of intervention activities during JC training workshops. Specifically, prompting participants to take the perspective of an agent in their workplace may be a preparatory step before participants engage in creating action plans which usually accompany JC training interventions [24]. Thus, in Study 2, we manipulated these two perspectives to test their effect on forming JC intentions. The data that support the findings of this study are openly available in Open Science Framework at https://osf.io/a6fp9/?view_only=7390b1210f224a209e762e5d6b670cc3.

### Method

#### Power analysis

We used G*power to estimate the appropriate sample size with 80% power and alpha of 0.05. The design was 3 (condition: agent, recipient, control) × 4 (JC intentions type: seeking structural resources, seeking social resources, increasing challenging job demands, decreasing hindering demands). The first variable was manipulated between subjects (i.e., each participant was randomized to only one condition) and the second was a within-subject variable (i.e., each participant reported on all four intentions). We expected a small interaction effect between these variables. The power analysis was conducted for within-between interaction in repeated-measures ANOVA with expected effect size of partial eta^2^ = 0.02 (f = 0.14). Based on previous meta-analyses concerning inter-relationship between JC dimensions [7], we assumed a low correlation between JC intention types (*r* = 0.20). A-priori sample size calculation estimated sample size of at least 144 subjects. We expected that some participants may not adhere to the task that introduced the manipulation, so we aimed to recruit at least 200 subjects.

#### Participants and procedure

The participants were recruited through an internal university database of employed students.^1^ We excluded 22 records of participants who did not follow the instructions in the experimental manipulation, turned out to be unemployed, or who had participated in a similar study in the past. We also excluded 5 participants who did not complete any of the scales. The final study sample (*N* = 216) consisted of 37 men and 179 women aged between 18 and 50 (*M*_age_ = 27, *SD* = 8).

The study was conducted online. The purpose of the research and respondents’ rights were presented on the first page of the survey. Then, a randomization procedure executed by a PHP script assigned participants to one of three conditions: with a manipulation activating the agent perspective (*N* = 64), the recipient perspective (*N* = 80), or to a control condition (*N* = 72). After manipulation check, the participants declared their JC intentions in the upcoming week.

### Materials and measures

#### Manipulation of perspectives

In the introduction, the respondents were informed that the discoveries of the Australian researchers showed that writing in a diary influences human well-being. Thus, the purpose of the presented study was to test whether this effect also occurs when a person rewrites the content of someone else’s diary. The respondents were asked to carefully read a diary note and transcribe it. The note described a schedule of entertainment for a free day. The story content was exactly the same in all conditions, but the perspective of the storyteller differed (see Additional file 3). In the agent’s condition, the participant had to rewrite a note indicating that they were the one who prepared this schedule for a friend (i.e., the storyteller was in charge). In the recipient’s condition, the note indicated that the plan was prepared for them by a friend (i.e., the story-teller had to succumb). In the control group, the note indicated that one person planned a day for another person, but the storyteller was neither of these two characters. A similar manipulation has been used in previous research with agent-recipient perspectives [10].

#### Manipulation control

To check the effectiveness of the manipulation, we used a four-item scale (e.g., “I felt I had no control over the situation”, “I had a capacity to act” [*α* = 0.72]) previously applied in a DPM manipulation [10]. The respondents used a 7-point scale (1 = definitely not, 7 = definitely yes).

#### Questionnaires

*Intention to craft* was measured with a modified version of the JC scale used in Study 1. Instead of reporting past behavior, participants reported their *intentions* to craft their jobs in the upcoming week (for similar procedure see Tims et al. [25]). An example item for increasing structural job resources is “I will try to develop myself professionally” (*α* = 0.87), for increasing social job resources is “I will ask others for feedback on my job performance” (*α* = 0.83), for increasing challenging job demands is “When an interesting project comes along, I will offer myself proactively as project co-worker” (*α* = 0.88), and for decreasing hindering job demands is “I will try to ensure that my work will be emotionally less intense” (*α* = 0.81). The participants indicated how often they intended to engage in each of the behaviors (1 = never, 5 = very often).

### Result and discussion

#### Analysis strategy

The differences in four types of JC intentions between the three experimental conditions were investigated by means of two-way mixed ANOVA. Mauchly’s test of sphericity was significant (*p* < 0.001), therefore sphericity assumption was not met. The approach with adjusted degrees of freedom was employed (Huynh–Feldt correction).

#### Manipulation control

First, we tested the effectiveness of manipulation by conducting a one-way ANOVA, which demonstrated substantial differences between conditions, *F* (2, 213) = 73.64, *p* < 0.001, *η*^2^ = 0.409. Levene’s test of equality of error variance was significant (*p* = 0.032), therefore pairwise comparisons were conducted using Tamhane T2. In the manipulated agent perspective (*M* = 5.18, *SD* = 1.19), participants felt substantially more in control of the situation than in the recipient perspective (*M* = 2.84, *SD* = 0.93; *d* = 2.04), and as compared to the control group (*M* = 3.91, *SD* = 1.33, *d* = 1.11), with large differences between the latter two (*d* = 0.93). All differences were statistically significant at *p* < 0.001. We conclude that the manipulation was effective.

#### Hypotheses testing

First, we observed a main effect of JC intentions, *F* (2,57, 548.13) = 80.44, *p* < 0.001, *η*_*p*_^*2*^ = 0.274, *η*^*2*^ = 0.164, ω^*2*^ = 0.167). Regardless of the experimental condition, participants intended to engage in increasing structural job resources more frequently (*M* = 4.05) than in other types of JC (*p*s < 0.001). Increasing social job resources (*M* = 2.90) was the least frequently planned among all types of JC (*p*s < 0.001). Increasing challenging job demands (*M* = 3.25) and decreasing hindering job demands (*M* = 3.14) did not differ significantly from each other. Second, the analysis revealed a main effect of the experimental condition, *F* (2, 213) = 3.80, *p* = 0.024, *η*_*p*_^2^ = 0.034, *η*^*2*^ = 0.013, ω^*2*^ = 0.009). Regardless of the type of JC, individuals in an agent perspective condition formed intentions to craft their jobs more frequently (*M* = 3.51) than individuals in a recipient perspective condition (*M* = 3.25; *p* = 0.046) but not in control group (*M* = 3.25;* p* = 0.053).

**Fig. 2:**
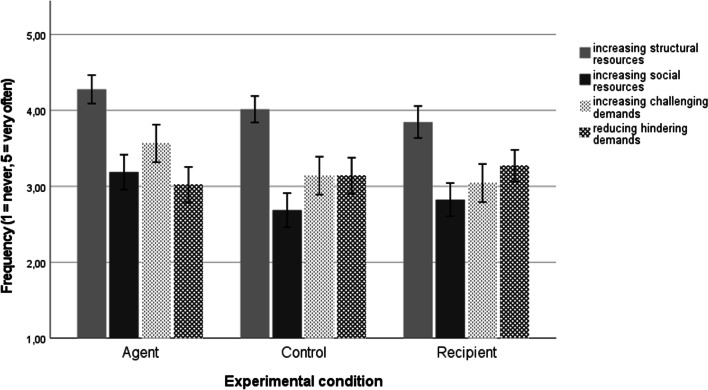
Job crafting intentions as a function of experimental condition. *Note.* The error bars indicate 95% confidence intervals

As expected, the analysis showed a significant interaction effect between the three conditions and the four types of crafting, *F* (5.15, 548.13) = 3.96, *p* = 0.001, *η*_*p*_^*2*^ = 0.036, *η*^*2*^ = 0.016, ω^*2*^ = 0.010). Post-hoc comparisons were conducted with Bonferroni correction for multiple comparisons. The results are presented in Fig. 2.

The pairwise analyses partially supported Hypothesis 1: activation of the agent perspective led to significantly stronger intentions to seek structural job resources as compared to the recipient perspective (*M* = 4.28, *SD* = 0.75 vs. *M* = 3.85, *SD* = 0.95, *p* = 0.006, *d* = 0.50). The effect size was medium. The difference between the agent perspective and the control group was small-to-medium in size but not statistically significant (*M* = 4.02, *SD* = 0.74, *p* = 0.200, *d* = 0.35).

Our findings partially supported Hypothesis 2: the agent perspective was positively linked with intentions to seek challenging job demands (*M* = 3.56, *SD* = 0.99) as compared to the recipient perspective (*M* = 3.04, *SD* = 1.13,* p* = 0.012, *d* = 0.49). The effect size was medium. The difference between the agent perspective and the control group was small-to-medium in size but non-significant (*M* = 3.14, *SD* = 1.06, *p* = 0.065, *d* = 0.41).

Contrary to Hypothesis 3, the recipient perspective was linked with significantly lower intentions to seek social job resources than the agent perspective, and this effect was small-to-medium in size (*M* = 2.83, *SD* = 0.98 vs. *M* = 3.19, *SD* = 0.92; *p* = 0.017, *d* = 0.38). While the recipient condition did not substantially differ from the control group (*M* = 2.69, *SD* = 0.96; *p* = 1.000, *d* = 0.14), there was a medium effect size difference between the agent group and the control group (*p* = 0.008, *d* = 0.53). This pattern is inconsistent with Hypothesis 3.

We observed a small insignificant difference between the agent vs. recipient perspective in terms of decreasing hindering job demands (*M* = 3.02, *SD* = 0.94 vs. *M* = 3.27, *SD* = 0.94, *p* = 0.366, *d* = 0.27). There were no substantial differences between these two groups (agent: *d* = 0.12 and recipient: *d* = 0.13; *p*s = 1.000) and the control condition (*M* = 3.14, *SD* = 1.01). Thus, Hypotheses 4 and Hypotheses 5 were not supported.

To summarize, we found mixed support for the influence of manipulated agent and recipient perspectives on JC intentions. As expected, the findings revealed that activating the agentic mindset increased participants’ intentions to seek more structural job resources and to increase their job challenges as compared to a recipient mindset; however, the differences from control group were small and non-significant. Participants did not differ in their intentions to reduce hindering demands. Finally, contrary to our expectations, activating an agentic mindset led to increased intentions to seek social job resources.

## Discussion

Past research has demonstrated that employees craft their jobs with different frequencies using distinct strategies. In this research we create a bridge between the DPM [8] and the JD-R [9] models to gain more insight into what kind of mindset predisposes people to craft their jobs and in what way. Below, we explain these contributions.

### Theoretical implications

The first contribution of this article is that by using the DPM model we were able to explain why some individuals have a greater tendency to modify job characteristics to fit their preferences. First, we showed that a chronic agent perspective is linked with approach crafting via increasing structural job resources and seeking challenging job demands. Agents believe that they are in control of their environment. In the workplace, this attitude may translate into perceiving oneself as an active contributor to the organizational reality. When those who view themselves as agents perceive that there is a misfit between actual and preferred job characteristics, they are more likely to ‘take charge’ and change unfavorable conditions. Additionally, forming job-crafting intentions is more likely for agents than for recipients. Thus, our research answers the call in the literature [3] to identify the type of mindset that is vital for developing JC intentions.

Simultaneously, we found that agents and recipients adopt different job redesign strategies. Thus, the second contribution of our research is a deeper understanding of patterns of JC strategies for agents and recipients that explain the orientation (level 1 in [4]) and content (Level 3 in [4]) of JC. As for the orientation, individuals who view themselves as agents are more likely to redesign their jobs in an approach-oriented way. Given the content, across two studies we showed that the agent perspective is linked with seeking structural job resources and increasing job challenges, as well as forming intentions to perform these behaviors in the future. The one expansion-oriented strategy that was not linked with agency in Study 1 was ‘seeking social job resources’. DPM predicts that agents are focused on goal pursuit and neglect communal aspects of the reality: research shows that goal activation inhibits activation of communal content [20]. This pattern of results, i.e., a tendency to increase structural job resources and challenging demands, but not social job resources, distinguishes the agent perspective from proactive personality, extraversion, or self-efficacy, which correlate with all expansion-oriented JC dimensions [7]. Thus, with this research we are introducing a unique predictor of certain job-crafting strategies.

As expected, a chronic tendency to adapt the recipient perspective was linked with reducing hindering job demands. DPM predicts that recipient perspective is related with a general contraction, i.e., behavioral withdrawal, vulnerability, and a lower tendency to take control. Thus, when job demands appear too taxing, recipients attempt to avoid them rather than face them. Our research shows that individuals with a chronic tendency to view themselves as ‘passive’ recipients of reality, tend to change their jobs by using avoidance crafting. This pattern puts the recipient perspective next to neuroticism or prevention focus as predictors of reducing hindering demands [7].

Despite our expectations, a recipient perspective was unrelated to seeking social job resources or forming intentions to engage in such behaviors in the future. Thus, while previous research has linked this perspective with focus on others and towards communal content [8], in our study this tendency did not translate into proactive behaviors related to seeking feedback or support at work. Possibly, the reactive aspects of the recipient perspective inhibit the pursuit of social resources. Thus, while recipients focus on others, their position is related to being dependent on them rather than showing the initiative towards them. This finding refines our understanding of DPM by demonstrating boundary conditions under which recipients focus on others. Because agent and recipient perspectives are orthogonal [18], future research should investigate an additive effect of chronic perspectives on increasing social job resources. Possibly, individuals with a strong recipient perspective engage in this crafting when their agency is also intensified.

Contrary to our expectations, Study 2 found that agents form intentions to seek social job resources to a higher extent than recipients, while agency was unrelated to this behavior in Study 1. This difference may result from the outcome that we tested: while Study 1 investigated past behaviors, Study 2 examined future intentions. Thus, though agents generally plan more approach-oriented crafting behaviors (regardless of their content), they do not, in fact, engage in looking for social job resources. This explanation should be further tested in future studies.

Importantly, in Study 1 we controlled proactivity, which had proven to be a relatively strong predictor of JC in an earlier literature reviews [3, 5]. We showed that the DPM explained additional variance in seeking structural job resources, seeking challenges, and decreasing hindering job demands. Furthermore, despite face value similarities between proactivity and agent perspective, we showed that these features explain differential patterns of JC. Specifically, proactivity was a positive predictor of increasing social job resources, while it was not the case for chronic agent perspective. In previous research [6] proactivity was a positive predictor of decreasing hindering job demands, while the agent perspective demonstrated to be a negative one. Thus, our research shows unique value in agent perspective as an antecedent of beneficial JC behaviors. This predictor is especially valuable since the agent perspective can be temporarily activated to prompt individuals to form JC intentions, while it does not increase a tendency to reduce job demands, which is a maladaptive crafting strategy [26].

Despite findings from Study 1, in Study 2 we were unable to detect the differences between agents and recipients in their intentions to reduce hindering job demands. One possible explanation may be a general reluctance of our study participants to form intentions about this type of behavior, given its negative connotations. Alternatively, avoidance crafting may be more reactive than planned, i.e., employees may resort to this when they are taxed; however, this is not a strategy that they premeditate. This explanation would be consistent with a meta-synthesis of JC research, which distinguished between proactive and reactive motives to craft [14]. The latter were often related to taxing context and served as coping strategies rather than proactively planned behaviors. In that vein, a recent intervention showed that those with high pre-intervention workload engaged in more crafting behaviours to decrease hindering demands, whereas those with low initial workload engaged in more crafting behaviours to increase structural resources [27].

### Practical implications

Constant changes that require fast adaptations make it difficult for organizations to create ideal job designs for each employee. Hence, it has become increasingly important for employees to take a proactive stance toward their jobs. Our research shows that the agentic mindset could be relevant for this problem. Thus, in the selection processes, organizations should value candidate’s propensities to an agentic mindset. Items from the Perspectives Questionnaire could serve as a basis for questions for the behavioral interview [28].

Our findings have implications for interventions aimed at boosting JC behaviors. The possibility to temporarily activate an agentic mindset and—by doing so—to foster generating JC intentions suggests a method that could be introduced during the action planning phase of such workshops. JC facilitators may use the DPM to help employees form JC goals. A task like the one we invented or is variation is a relatively quick, simple, and free activity that can become a part of a JC intervention, both during workshops [29] and online interventions[30]. Additionally, some JC interventions use reminders or boosters to make intervention effects sustainable after the workshop phase is finished [27, 29]. Our experimental study shows that short exercises based on DPM could potentially nudge individuals towards more crafting. Similarly, managers can play an active role in motivating individuals to undertake approach crafting by empowering them to feel more in control of their job design by recalling certain situation where they were more agentic. Team reflexivity interventions have proven successful as sources of bottom-up job redesign among employees by increasing their agency [31].

Additionally, because previous findings demonstrated that agency is linked with increased experience of control, self-efficacy, and self-esteem[8], our study shows that the activity introduced in our experimental study could be used as an easy and effective method to activate an agentic mindset as a way to boost personal resources in interventions in other areas where personal resources might be relevant, such as education or changing health behaviors.

### Limitations and future directions

Certain limitations need to be acknowledged. First, Study 1 applied a cross-lagged research design and used self-reports. Thus, our data was potentially subject to common method bias in T1 [32]. To test if this was the case, we performed CFAs on T1 variables (see Additional file 1), which showed that the variance in our data could not be attributed to a single factor since a multi-factor solution fits the data better. This result indicates that common method bias is not a major issue in this study. Yet, while the seven-factor model fit the data best, the final fit indices were suboptimal compared to recommendations. These estimates could, however, result from violated multinormality assumptions and chosen response scales.

Another limitation relates to the operationalization of the recipient perspective in the Perspectives Questionnaire [18] that we applied in Study 1. Closer examination of the items may lead to the conclusion that this subscale measures social orientation or emotional intelligence rather than a chronic tendency to think of oneself as a recipient ‘awaiting actions’ from others in the social world. While these two aspects (orientation to communion and focus on one’s emotions) are important aspects of the construct, we believe that the instrument could be further revised to incorporate the contraction/withdrawal of action dimension more. Consequently, we treat studies 1 and 2 more as conceptual than direct replications.

Our experiment showed that activating an agent perspective led to stronger intentions to increase structural resources and job challenges as compared to the recipient perspective; yet, when comparing to the control group, the effect sizes were small-to-medium and statistically insignificant. However, in agent-recipient framework a control group may be controversial, as in the regular dynamic there are only two roles: one who is the author of an action and the other one who is its recipient. In fact, in a series of experimental studies where these perspectives were manipulated in different ways, Bialobrzeska et al. [10] did not introduce a control condition. Future researchers could apply a pre-post design to observe an actual increase in intentions to craft one’s job after activating the agent perspective. Additionally, we suggest that a new agent-recipient manipulation may be designed that more strongly relates to the work contexts.

Because the two samples were obtained from a university participant pool, the conclusions are limited to employed adults with university experience. In addition, the majority of the sample was female, which could affect both the propensity to take agent–recipient perspectives [18], as well as engage in JC [7]. Specifically, past research shows that women are more likely to assume the recipient perspective than men [18]. Hence, future research should aim for more balanced samples. Moreover, our research was conducted in only one cultural setting (Poland). Yet, the tendency to take an agent or recipient perspective may differ between countries as a function of, e.g., masculinity-femininity dimensions [33]. Poland is a more masculine society compared to e.g., Scandinavian countries. Thus, in other contexts it may be more difficult to activate agency as means of prompting job crafting intentions. Additionally, Poland scores high on the ‘uncertainty avoidance’ which translates into creating procedures and rules, and intolerance for deviant behavior. Thus, job crafting may be less socially approved there than in the Netherlands or Sweden. To generalize our findings, the observed relationships should be examined in distinct cultural settings as a function of the distinguished dimensions.

## Conclusions

This article presents the results of two studies that investigated the link between agent-recipient perspectives and JC. By integrating DPM [8] with the JD-R [9] model we aimed to explain *who* engages in JC and *in what form*. We demonstrated that agents are more likely to seek structural job resources and challenging job demands, while recipients tend to reduce hindering job demands. We also showed that activation of agent perspectives helps participants form JC intentions, which is relevant for workplace interventions on proactivity.

## Supplementary Information

**Additional file 1:** Confirmatory Factor Analysis

**Additional file 2:** Table A. Means (M), Standard Deviations (SD), and Correlations Between Proactivity, Agent, Recipient and Job Crafting in Study 1

**Additional file 3:** Perspective manipulation in Study 2

## Data Availability

The data that support the findings of Study 1 and 2 are openly available in Open Science Framework at https://osf.io/a6fp9/?view_only=7390b1210f224a209e762e5d6b670cc3.

## Abbreviations

DPM: Dual Perspective Model
JC: Job crafting
JD-R: Job Demands-Resources
T1: Time point one
T2: Time point two

## Footnotes

1 Participants from the two studies did not overlap.
