# Supplementary material for: It all depends on which side of the fence you are standing: agent and recipient perspectives are differently linked with job crafting
Source: BMC Psychol. 2023 Apr 4;11:98. doi: 10.1186/s40359-023-01135-0 (PMC10074650; doi:10.1186/s40359-023-01135-0)
Supplement: Supplementary file 1 — Additional file 1: Confirmatory Factor Analysis [file 40359_2023_1135_MOESM1_ESM.docx]

**Additional file 1 – supplementary material to**

**It All Depends on Which Side of the Fence You Are Standing: Agent and Recipient Perspectives Are Differently Linked With Job Crafting**

**Confirmatory Factor Analysis**

To test the construct validity of the measures, we conducted a series of confirmatory factor analyses (CFA) in Jamovi [1]. The proposed model assumed seven factors: proactivity, agent perspective, recipient perspective, increasing structural job resources, increasing social job resources, increasing challenging job demands, and decreasing hindering job demands. This structure was compared to two alternative solutions by means of testing the change in χ2 values: (1) a one-factor model with all items loading onto one factor and (2) a four-factor model with proactivity, agent perspective, recipient perspective, and all job crafting dimensions as one factor. In line with the multifaceted approach to assessment of model fit, we considered the following fit indices: Comparative Fit Index (CFI; [2], Tucker and Lewis Index (TLI;[3], Root Mean Square Error of Approximation (RMSEA; [4]), along with 90 % confidence interval (CI) limits, and (Standardized) Root Mean Square Residual ([S]RMR; [2]. We used the following values as thresholds recommended in the literature: TLI and CFI > .90, RMSEA < .08, and (S)RMR < .08. We followed the steps described for T1 data.

The results indicated that the proposed 7-factor model (proactivity, agent perspective, recipient perspective, increasing structural job resources, increasing social job resources, increasing challenging job demands, and decreasing hindering job demands) fit the data reasonably well: χ^2^ = 2490, df = 1154, RMSEA = .06 [90% CI: .06―.06], CFI = .80, TLI = .79, SRMR = .07. This measurement model was superior (Δ*χ*^2^ = 879, Δdf = 15, *p* < .001) to a 4-factor model (proactivity, agent perspective, recipient perspective, and all job crafting dimensions as one factor): χ^2^ = 3369, df = 1169, RMSEA = .08 [90% CI: .07―.08], CFI = .67, TLI = .65, SRMR = .09. This model also fit the data better (Δ*χ*^2^ = 2829, Δdf = 21, *p* < .001) than a 1-factor model χ^2^ = 5319, df = 1175, RMSEA = .10 [90% CI: .10―.11], CFI = .38, TLI = .35, SRMR = .12.

Overall, while the 7-factor model fits the data best, the CFI and TLI indices’ values are suboptimal comparing them to the recommendations from the literature[5]. In CFA, as part of the structural equation modeling methods family, application of the RMSEA, CFI, and TLI highly relies on the conventional cutoff values developed under normal-theory maximum likelihood (ML) with continuous data [6]. We tested the multinormality assumptions and observed that, indeed, this assumption is violated (significant multivariate skew and kurtosis tests). Additionally, Likert scales, which we applied in this project, could be considered ordinal data, especially if the number of response categories is lower than 10 [7]. In that case, unweighted least squares (ULS) and diagonally weighted least squares (DWLS) estimation methods could be applied to test model fit. Recent simulations studies suggest that these estimation methods have substantial impacts of the indices’ values, and different cut-off values could be employed [6, 8].

Therefore, we additionally tested the model fit of the 7-factor model using ULS and DWLS estimators. Because jamovi only uses ML estimator, this supplementary analysis was performed in JASP [9]. The results showed higher CFI/TLI indices for these estimators (respectively): .90/.89 for ULS and .95/.94 for DWLS.

**References**

1. The jamovi project. No Title. Jamovi (Version 0.9) [Computer Software]. 2019.

2. Bentler PM. Comparative fit indexes in structural models. Psychol Bull. 1990;107:238–46.

3. Bagozzi RP, Yi Y. On the evaluation of structural equation models. J Acad Mark Sci. 1988;16:74–94.

4. Browne MW, Cudeck R. Alternative ways of assessing model fit. Sociol Methods Res. 1992;21:230–58.

5. Hu L, Bentler PM. Cutoff criteria for fit indexes in covariance structure analysis: Conventional criteria versus new alternatives. https://doi.org/101080/10705519909540118. 2009;6:1–55.

6. Xia Y, Yang Y. RMSEA, CFI, and TLI in structural equation modeling with ordered categorical data: The story they tell depends on the estimation methods. Behavior Research Methods 2018 51:1. 2018;51:409–28.

7. Wu H, Leung S-O. Can Likert Scales be Treated as Interval Scales?—A Simulation Study. http://dx.doi.org/101080/0148837620171329775. 2017;43:527–32.

8. Shi D, Maydeu-Olivares A. The Effect of Estimation Methods on SEM Fit Indices: https://doi.org/101177/0013164419885164. 2019;80:421–45.

9. JASP Team. JASP (0.14.1). 2021.
