# Supplementary material for: It all depends on which side of the fence you are standing: agent and recipient perspectives are differently linked with job crafting
Source: BMC Psychol. 2023 Apr 4;11:98. doi: 10.1186/s40359-023-01135-0 (PMC10074650; doi:10.1186/s40359-023-01135-0)
Supplement: Supplementary file 2 — Additional file 2: Table A. Means (M), Standard Deviations (SD), and Correlations Between Proactivity, Agent, Recipient and Job Crafting in Study 1 [file 40359_2023_1135_MOESM2_ESM.docx]

**Additional file 2 – supplementary material to**

**It All Depends on Which Side of the Fence You Are Standing: Agent and Recipient Perspectives Are Differently Linked With Job Crafting**

**Table A**

| *Means (M), Standard Deviations (SD), and Correlations Between Proactivity, Agent, Recipient and Job Crafting in Study 1* | | | | | | | | | | | | | | | | | | | | |  | |  | |  | |  |
| --- | --- | --- | --- | --- | --- | --- | --- | --- | --- | --- | --- | --- | --- | --- | --- | --- | --- | --- | --- | --- | --- | --- | --- | --- | --- | --- | --- |
|  | | | |  |  | |  | |  | |  | |  | |  | |  | |  | |  | |  | |  | |  |
| **T1-T1 (*N* = 324)** | ***M*** | ***SD*** | **P T1** | | | **A T1** | | **R T1** | | **IStR T1** | | **ISoR T1** | | **IC T1** | | **RHD T1** | | **-** | | **-** | | **-** | | **-** | |  |  |
| Proactivity, T1 (P T1) | 5.06 | 0.92 |  | | |  | |  | |  | |  | |  | |  | |  | |  | |  | |  | |  |  |
| Agent, T1 (A T1) | 5.20 | 0.71 | .61*** | | |  | |  | |  | |  | |  | |  | |  | |  | |  | |  | |  |  |
| Recipient, T1 (R T1) | 5.12 | 0.98 | .08 | | | .07 | |  | |  | |  | |  | |  | |  | |  | |  | |  | |  |  |
| Increasing Structural Resources, T1 (IStR T1) | 3.94 | 0.67 | .47*** | | | .46*** | | .01 | |  | |  | |  | |  | |  | |  | |  | |  | |  |  |
| Increasing Social Resources, T1 (ISoR T1) | 2.71 | 0.83 | .19*** | | | .07 | | .07 | | .23*** | |  | |  | |  | |  | |  | |  | |  | |  |  |
| Increasing Challenges, T1 (IC T1) | 3.09 | 0.90 | .38*** | | | .38*** | | -.001 | | .56*** | | .40*** | |  | |  | |  | |  | |  | |  | |  |  |
| Reducing Hindering Demands, T1 (RHD T1) | 2.90 | 0.71 | .09 | | | -.04 | | .29*** | | -.12* | | .07 | | -.15** | | - | |  | |  | |  | |  | |  |  |
| **T1-T2 (*N* = 146)** | ***M*** | ***SD*** | **P T1** | | | **A T1** | | **R T1** | | **IStR T1** | | **ISoR T1** | | **IC T1** | | **RHD T1** | | **IStR T2** | | **ISoR T2** | | **IC T2** | | **RHD T2** | |  |  |
| Proactivity, T1 (P T1) | 5.05 | 0.95 |  | | |  | |  | |  | |  | |  | |  | |  | |  | |  | |  | |  |  |
| Agent, T1 (A T1) | 5.22 | 0.72 | .57*** | | |  | |  | |  | |  | |  | |  | |  | |  | |  | |  | |  |  |
| Recipient, T1 (R T1) | 5.03 | 1.06 | .08 | | | .14 | |  | |  | |  | |  | |  | |  | |  | |  | |  | |  |  |
| Increasing Structural Resources, T1 (IStR T1) | 3.95 | 0.71 | .50*** | | | .44*** | | -.04 | |  | |  | |  | |  | |  | |  | |  | |  | |  |  |
| Increasing Social Resources, T1 (ISoR T1) | 2.67 | 0.82 | .30*** | | | .16 | | .07 | | .38*** | |  | |  | |  | |  | |  | |  | |  | |  |  |
| Increasing Challenges, T1 (IC T1) | 3.15 | 0.93 | .50*** | | | .44*** | | -.04 | | .71*** | | .50*** | |  | |  | |  | |  | |  | |  | |  |  |
| Reducing Hindering Demands, T1 (RHD T1) | 2.89 | 0.70 | .15 | | | .01 | | .29*** | | -.17* | | .13 | | -.11 | |  | |  | |  | |  | |  | |  |  |
| Increasing Structural Resources, T2 (IStR T2) | 3.97 | 0.68 | .44*** | | | .37*** | | -.04 | | .61*** | | .24** | | .53*** | | -.07 | |  | |  | |  | |  | |  |  |
| Increasing Social Resources, T2 (ISoR T2) | 2.72 | 0.78 | .35*** | | | .15 | | .09 | | .26** | | .64*** | | .36*** | | .04 | | .33*** | |  | |  | |  | |  |  |
| Increasing Challenges, T2 (IC T2) | 3.09 | 0.96 | .38*** | | | .34*** | | -.01 | | .58*** | | .37*** | | .73*** | | -.12 | | .64*** | | .45*** | |  | |  | |  |  |
| Reducing Hindering Demands, T2 (RHD T2) | 2.92 | 0.70 | .07 | | | -.10 | | .28** | | -.19* | | .18* | | -.08 | | .61*** | | -.23** | | .12 | | -.12 | | - | |  |  |
|  | | | |  |  | |  | |  | |  | |  | |  | |  | |  | |  | |  | |  | |  |
| *Note. N*_T1_ = 324; *N*_T2_ = 146 | | | | | | | | | | | | | | |  | |  | |  | |  | |  | |  | |  |
| *** *p* < .001, ** *p* < .01, * *p* < .05 | | | | | | | | | | |  | | | |  | |  | |  | |  | |  | |  | |  |
