# Supplementary material for: It all depends on which side of the fence you are standing: agent and recipient perspectives are differently linked with job crafting
Source: BMC Psychol. 2023 Apr 4;11:98. doi: 10.1186/s40359-023-01135-0 (PMC10074650; doi:10.1186/s40359-023-01135-0)
Supplement: Supplementary file 3 — Additional file 3: Perspective manipulation in Study 2 [file 40359_2023_1135_MOESM3_ESM.docx]

**Additional file 3 – supplementary material to**

**It All Depends on Which Side of the Fence You Are Standing: Agent and Recipient Perspectives Are Differently Linked With Job Crafting**

**Perspective manipulation in Study 2** (based on manipulation used in Bialobrzeska et al., 2019; Study 1).

*Here, we present translated research material. Original instructions (in Polish) can be found below. Participants were randomly assigned to one of the three conditions: agent, recipient, or control.*

(Cover story: all conditions)

The latest discoveries of Australian researchers showed that expressive writing about one’s experiences in a diary influences human well-being. The purpose of this study is to test whether this effect also occurs when a person rewrites the content of someone else’s diary, i.e., adopts the “I” perspective and writes about something that he or she did not experience him- or herself.

You have just written a note in your diary and now you are reading it. Please, read the following text carefully and rewrite it in the box below. Imagine that you are the author – GET INTO THE ROLE OF THE AUTHOR OF THE TEXT, BUT DO NOT MODIFY IT.

(Agent)

I have just finished planning the Saturday for Marysia - my friend. We have a tradition that from time to time we plan Saturdays for each other, and then the planning person has an influence on how the day looks like. Today I am planning, so everything depends on me; this is our rule. The weather is nice, so I decided that at 12:00 we will go for a walk to the beach. After the walk, I planned a coffee at our favorite cafe by the pier. Then, I decided that we will go to the shopping center to choose a watch. I decided that we will have dinner in a Mexican restaurant. I planned that we would be back home about 19:00. It will definitely be fun.

(Recipient)

Marysia - my friend - has just finished planning the Saturday for me. We have a tradition that from time to time we plan Saturdays for each other, and then the planning person has an influence on how the day will look like. Today Marysia is planning, so everything depends on her; this is our rule. The weather is nice, so Marysia decided that at 12:00 we will go for a walk to the beach. After the walk, she planned a coffee at our favorite cafe by the pier. Then, she decided that we will go to the shopping center to choose a watch. Marysia decided that we will have dinner in a Mexican restaurant. She planned that we would be back home about about 19:00. It will definitely be fun.

(Control condition)

Marysia and Kasia have a tradition that they plan each other Saturdays from time to time, and then the planning person has an influence on how the day will look like. Today Marysia is planning, so everything depends on her; this is their rule. It is beautiful day, so Marysia decided that at 12:00 they will go for a walk to the beach. After the walk, she planned a coffee at their favorite cafe by the pier. Then, she decided to go to the shopping center to choose a watch. Marysia decided that they will have dinner in a Mexican restaurant. She planned that they would be back home about 19:00. It will definitely be fun.

| *Original instructions in Polish* |
| --- |

Ostatnie badania australijskich naukowców pokazały, że ekspresyjne pisanie w pamiętniku o własnych przeżyciach wpływa na dobrostan człowieka. W tym badaniu chcemy sprawdzić, czy efekt ten pojawi się również wtedy, gdy ludzie będą pisali z perspektywy "ja"? Czyli będą wczuwali się w postać autora, ale jednocześnie nie będą pisali o czymś, co realnie sami przeżyli.

Czytasz w swoim pamiętniku to, co przed chwilą w nim napisałeś/-aś. Przeczytaj proszę uważnie, a następnie przepisz poniższy tekst, wyobrażając sobie, że jesteś jego autorem – POSTARAJ SIĘ MOCNO WCZUĆ W ROLĘ AUTORA TEKSTU, ALE PRZEPISUJĄC NIE MODYFIKUJ GO. Jednakże dostosuj końcówki do swojej płci.

(Sprawca)

Kończę właśnie planować sobotę dla Marysi – mojej przyjaciółki. Mamy taki zwyczaj, że od czasu do czasu planujemy sobie wzajemnie soboty i wtedy osoba planująca ma wpływ na to, jak ten dzień będzie wyglądał. Dziś ja planuję, a więc wszystko zależy ode mnie; taką mamy umowę. Jest piękna pogoda, więc postanowiłem/-am, że o godzinie 12:00 pójdziemy na spacer nad morze. Po spacerze zaplanowałem/-am kawę w naszej ulubionej kawiarni przy molo. Następnie zadecydowałem/-am, że podskoczymy do centrum handlowego, żeby obejrzeć zegarek Postanowiłem/-am, że obiad zjemy w restauracji meksykańskiej. Powrót zaplanowałem/-am na około 19:00. Na pewno będzie fajnie.

(Biorca)

Marysia – moja przyjaciółka – skończyła właśnie planować sobotę dla mnie. Mamy taki zwyczaj, że od czasu do czasu planujemy sobie wzajemnie soboty i wtedy osoba planująca ma wpływ na to, jak ten dzień będzie wyglądał. Dziś Marysia planuje, a więc wszystko zależy od niej; taką mamy umowę. Jest piękna pogoda, więc Marysia postanowiła, że o godzinie 12:00 pójdziemy na spacer nad morze. Po spacerze zaplanowała kawę w naszej ulubionej kawiarni przy molo. Następnie zadecydowała, że podskoczymy do centrum handlowego, żeby obejrzeć zegarek. Marysia postanowiła, że obiad zjemy w restauracji meksykańskiej. Powrót zaplanowała na około 19:00. Na pewno będzie fajnie.

(Kontrolna)

Marysia i Kasia mają taki zwyczaj, że od czasu do czasu planują sobie wzajemnie soboty i wtedy osoba planująca ma wpływ na to, jak ten dzień będzie wyglądał. Dziś Marysia planuje, a więc wszystko zależy od niej; taką mają umowę. Jest piękna pogoda, więc Marysia postanowiła, że o godzinie 12:00 pójdą na spacer nad morze. Po spacerze zaplanowała kawę w ich ulubionej kawiarni przy molo. Następnie zadecydowała, że podskoczą do centrum handlowego, żeby obejrzeć zegarek. Marysia postanowiła, że obiad zjedzą w restauracji meksykańskiej. Powrót zaplanowała na około 19:00. Na pewno będzie fajnie.
